# Successful Treatment of a Case of Desmoplastic Fibroma: A Case of Unusual Lesion

**DOI:** 10.7759/cureus.17857

**Published:** 2021-09-09

**Authors:** Abdullah A Abu Alnasr, Sarmad R Sulaiman, Abdulaziz A Abu Alnasr, Yousef Qari, Rayan M Al Arabi

**Affiliations:** 1 Orthopedics, King Fahad Hospital, Medina, SAU; 2 Orthopedic Oncology, Madinah General Hospital, Medina, SAU; 3 Pediatrics, Prince Mohammed Bin Abdulaziz National Guard Hospital, Medina, SAU; 4 Neurology, King Abdullah Medical Complex, Jeddah, SAU

**Keywords:** non‑metastatic primary bone tumor, unusual lesion, orthopedic oncology, surgery, tumor, desmoplastic fibroma

## Abstract

Desmoplastic fibroma (DF) is a non-metastatic primary bone tumor that is extremely rare with local aggressive behavior. To the best of our knowledge, only few cases were published discussing this type of tumor and its management. This case report aimed to discuss a novel case as well as its management scheme. We present a case of a 36-year-old male with DF lesion involving the proximal tibial who underwent an extended curettage, triple type of adjuvant thereby, and internal fixation.

## Introduction

Desmoplastic fibroma (DF) is a non‑metastatic primary bone tumor extremely rare, lytic with local aggressive behavior with a prevalence of 0.1-0.3% is the prevalence of this tumor among all bone neoplasms [1]. DF was first described by Jaffe in 1958 [2]. DF may involve any bone. However, it is most often found in the mandible (22%) and less frequently in the femur (15%), pelvic bones (13%), radius (12%), and tibia (9%) [3,4]. Most published cases are below the age of 30 years. There is no gender predilection of this neoplasm and only 6% of the cases above the age of 50 years can be affected. The factors that were claimed to cause DF include trauma, endocrine, and genetic factors yet the exact causative factor is still not completely known [3]. Patients with DF suffer from pain or swelling for an extended period that comes before the functional disability. Less commonly, the initial picture of the clinical presentation of DF can be patients who show up with pathological fractures [5]. Whorled, creamy-colored tumors are the microscopic features of DF. In addition, low cellularity formed by bland of spindle cells in a collagenous matrix, limited mitoses, absence of necroses and presence of fibroblasts, myofibroblasts, and undifferentiated mesenchymal cells as the primary types of cells [6]. Several imaging features of DF are displayed in the following manner: usually well-defined or partly well-defined, narrow transition zone, lobulated form, expansile growth and sometimes with cortical bone destruction and extension into the adjacent tissues osteolytic or mixed osteolytic/mildly sclerotic matrix, well-defined or partly well-defined margins, possible endosteal scalloping or cortical breakthrough, geographic pattern of bone destruction, often non-sclerotic margins, internal pseudotrabeculation ( >90%), no matrix mineralization, and widening of the host bone from gradual apposition of periosteal new bone formation 90%. All of which are characteristics of DF on plain radiograph [6]. MRI stands as the modality of choice in visualizing soft tissue expansion. Besides the presence of the solid component, the existence of cystic changes might occur [6].

## Case presentation

A 36-year-old male, otherwise healthy, presented to the clinic complaining of left knee pain for eight months, which has been increasing recently with no history of trauma or constitutional symptoms, no knee locking, and no significant past surgical or medical history. Upon examination, the patient had a normal gait and upon inspection, the patient had mild anteromedial proximal tibial swelling firm to palpation. The skin was intact with no discoloration or engorged veins. Upon palpation, he had mild anteromedial proximal tibial tenderness. Arch of range of motion was from zero degrees full extension up to 120 flexions, no locking or instability, and an intact distal neurovascular examination.

**Figure 1:**
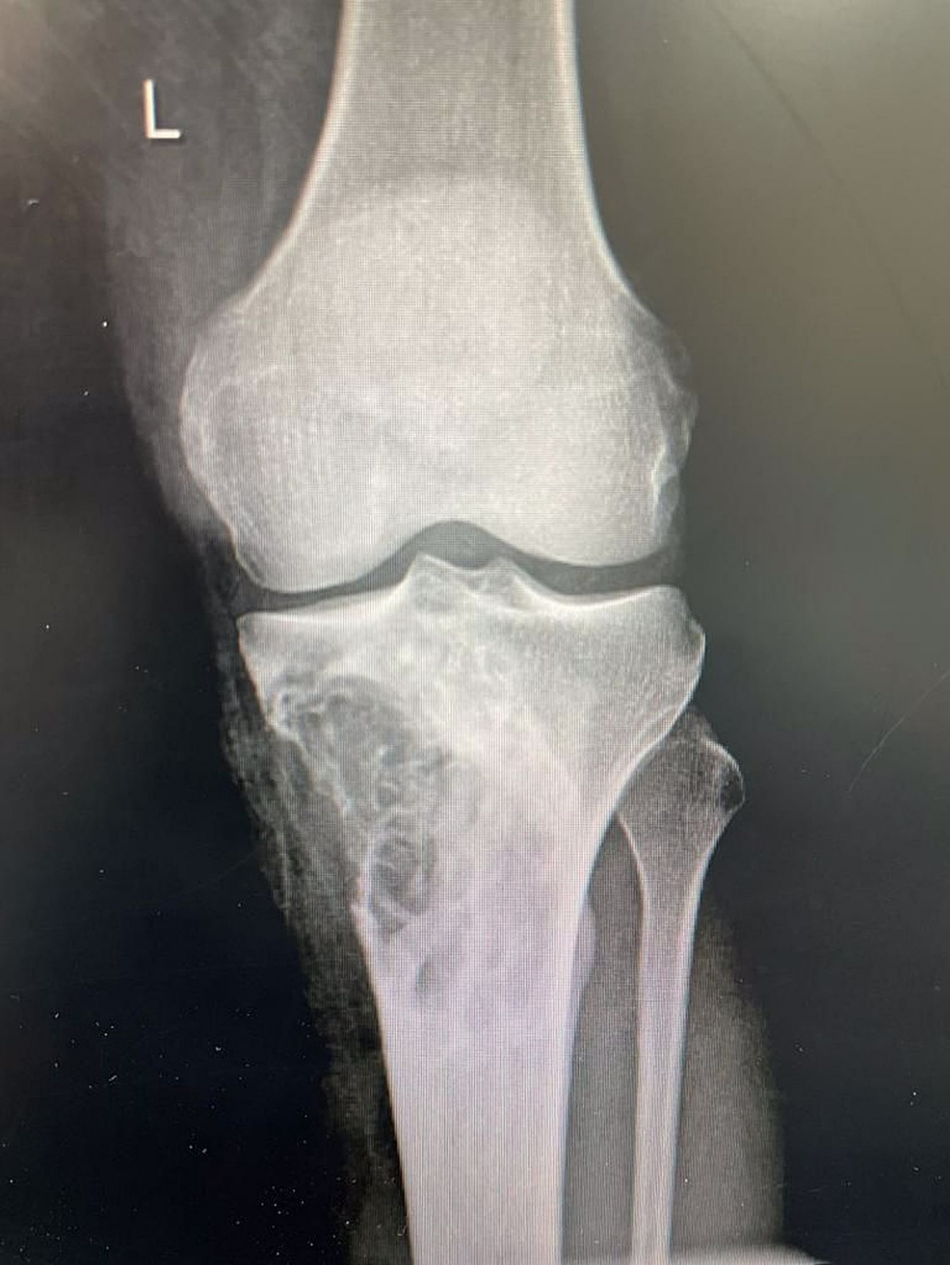
Pre-operative x-rays showing mix osteolytic osteosclerotic lesion involving the proximal tibial metaphysioepiphyseal with a narrow zone of transition and multiple lobulations, also a cortical breakthrough.

**Figure 2:**
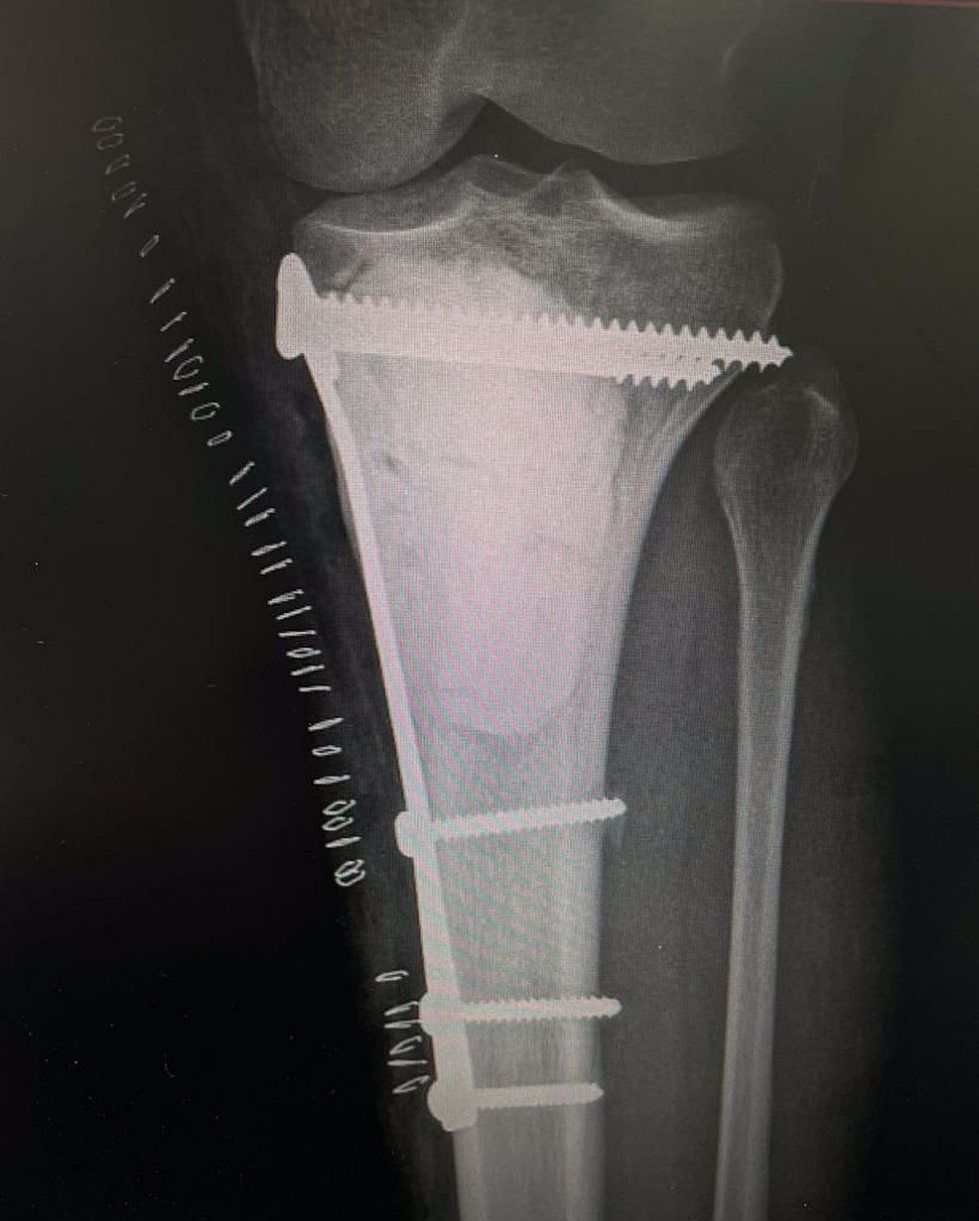
Post-operative x-rays showing the bone which was filled with cement after supporting the subchondral surface by an allograft layer of bony chips and fixed with a supportive medial side plate.

X-ray was done which showed a mix of osteolytic osteosclerotic lesion involving the proximal tibial metaphysioepiphyseal with a narrow zone of transition, multiple lobulations, and cortical breakthrough. Differential diagnosis at the beginning included aggressive benign tumor, malignant bone tumor, which could not be ruled out completely. We started with giant cell tumor, chondromyxoid fibroma, and desmoplastic fibroma. The systemic staging was done for the patient that included x-rays for the whole tibia as well as MRI. MRI showed well-defined eccentric proximal tibial metaphyseal lesion medially measuring about 5x5.4x7.5 cm in anteroposterior diameter (AP), transverse, and intercommissural diameter (CC). It was heterogeneous low signal intensity on T1 with few areas of high signal foci anteriorly likely representing hemorrhage. Heterogeneous high signal intensity on T2 peripherally with central low signal intensity and small cyst posteriorly measuring about 2x2.21x2.6 cm AP, transverse, and CC diameter, respectively. Intense enhancement is appreciated, this lesion of narrow zone transition, with no aggressive periosteal reaction, and no chondroid matrix can be identified. Cortical breakthrough at the medial aspect was noted, no perilesional edema was seen. The above-described lesion at that time could have been chondromyxoid fibroma, a giant cell tumor with secondary ABC changes. Thus, further evaluation with guided biopsy was recommended (Figures 1, 2).

**Figure 3:**
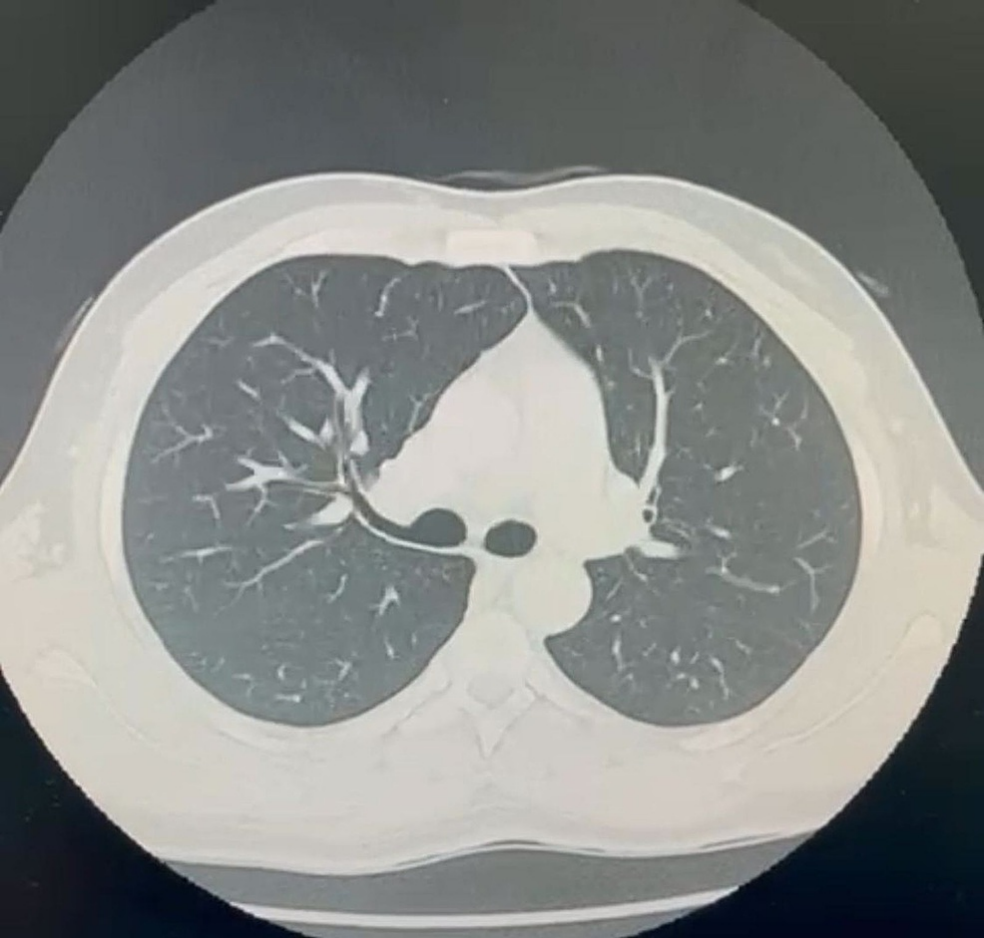
CT scan of the chest showing no metastasis to the thoracic cavity with overall normal appearance and vasculature.

Furthermore, a bone scan was done which showed a well-defined big area of a mixed pattern of abnormally increased uptake mixed with cold areas, maybe osteoclastic or osteoblastic lesions, involving the upper part of the left tibia. No other definitive area of abnormally increased uptake was seen. CT chest was done which showed no metastasis to the chest or upper abdomen. CT-guided biopsy was done for the patient and the histopathology report was suggestive of DF (Figure 3).

Subsequently, the patient was taken for hard extended curettage after exteriorization window. Then, mechanical adjuvant in form of curette and high-speed burr was used, thermal adjuvant inform of cautery was used, and chemical adjuvant inform of hydrogen peroxide was used. After that the subchondral surface was supported by allograft layer of bone chips, the rest of the defect was filled with cement and fixed by a supportive medial side plate. The patient was discharged on the second day of post-surgery. As tolerated, the patient was started on full weight-bearing immediately. Then the patient proceeded to do muscle strengthening exercises. Eventually, range of motion workouts with physical therapy were added to the rehabilitation regimen. The patient was followed for one year and a half with no symptoms, range of motion is full with no limitations in physical activities.

## Discussion

Desmoplastic fibroma (DF) is a non‑metastatic primary bone tumor that is extremely rare in nature, lytic with local aggressive behavior [7]. This patient had chronic knee pain with no predisposing factors. After a thorough study of medical history, physical examination, and proper investigations, the patient was diagnosed with DF that involved the left side of the proximal tibia, which was not the most common site of this tumor. According to the available literature, distinguishing between low-grade fibrosarcoma and DF is a huge challenge due to the overlap of both tumors that merge imperceptibly with each other; this poses a significant challenge even for the most experienced pathologists [7,8]. Image‑guided percutaneous biopsy has been adopted as the modality of choice in terms of the initial diagnosis for most musculoskeletal tumors. The complication rate of percutaneous needle biopsies is 1.1%, which is remarkably low when compared to the open biopsy complication rate of 16% [9]. Numerous treatment modalities have been proposed for the management of DF [10]. These involve curettage, wide resection with or without bone graft, cryosurgery, and amputation in patients with recurrence [7]. Although DF does not metastasize to other areas of the body, recurrence can happen in cases with incomplete removal of the neoplasm [5]. Higher recurrence rates are significantly observed in patients who are treated with simple curettage rather than wide surgical excision [10]. In our case, we chose to go for an extended curettage over simple curettage with the use of adjuvant and bone cement to save the patient's native need as a wide resection and proximal tibia replacement would be the aggressive way to manage in such case, age, and presentation.

## Conclusions

In this case, due to the tumor type, size, site, aggressive behavior, possible effect over the surrounding structures, and its impact on the range of motion, extended curettage after exteriorization window was performed with three types of adjuvants. This technique helped us achieve complete resection of the tumor. The patient was pain-free after surgery, and he was able to perform a full range of motion after a six-week physical therapy program, all of which reduced the patient's bed-bound time and hospital stay. The patient was discharged on the second day that can be attributed to the type of the utilized technique.
